# Age-dependent differences in learning to control a robot arm using a body-machine interface

**DOI:** 10.1038/s41598-018-38092-3

**Published:** 2019-02-13

**Authors:** Rajiv Ranganathan, Mei-Hua Lee, Malavika R. Padmanabhan, Sanders Aspelund, Florian A. Kagerer, Ranjan Mukherjee

**Affiliations:** 1 Department of Kinesiology, Michigan State University, East Lansing, USA; 2 Department of Mechanical Engineering, Michigan State University, East Lansing, USA; 3 Neuroscience Program, Michigan State University, East Lansing, USA

## Abstract

Body-machine interfaces, i.e. interfaces that rely on body movements to control external assistive devices, have been proposed as a safe and robust means of achieving movement and mobility; however, how children learn these novel interfaces is poorly understood. Here we characterized the learning of a body-machine interface in young unimpaired adults, two groups of typically developing children (9-year and 12-year olds), and one child with congenital limb deficiency. Participants had to control the end-effector of a robot arm in 2D using movements of the shoulder and torso. Results showed a striking effect of age - children had much greater difficulty in learning the task compared to adults, with a majority of the 9-year old group unable to even complete the task. The 12-year olds also showed poorer task performance compared to adults (as measured by longer movement times and greater path lengths), which were associated with less effective search strategies. The child with congenital limb deficiency showed superior task performance compared to age-matched children, but had qualitatively distinct coordination strategies from the adults. Taken together, these results imply that children have difficulty learning non-intuitive interfaces and that the design of body-machine interfaces should account for these differences in pediatric populations.

## Introduction

As the number of children needing assistive devices for mobility and manipulation increases^1^, the need for developing effective methods for the control of assistive devices such as a prosthetic arm remains a priority. Although substantial advances have been made in the area of brain-machine interfaces (BMI) to control high degree-of-freedom (DOF) assistive devices^2–4^, they are often not well suited for children. This is because invasive BMIs (such as microelectrode arrays) involve surgical risks and have limited longevity, whereas non-invasive BMIs (such as EEG) suffer from low signal-to-noise ratios, non-stationarity and can be cumbersome to wear for long periods of time^5,6^. As a result, there still remains a critical need for developing a robust interface for controlling high DOF assistive devices (e.g., a robot arm) for children.

An alternative to BMIs that has recently gained traction is the ‘body-machine’ interface (BoMI)^7^. In contrast to brain-machine interfaces, BoMIs rely on picking up existing body movements to control external devices. Because these movements can be picked up using small wireless devices (such as inertial measurement units), they possess a number of advantages including being completely non-invasive, having good signal properties (i.e. high signal-to-noise ratios and low drift), and being easily wearable. These BoMIs have been used in a wide variety of contexts, including spinal cord injury^8–11^, stroke^12,13^, and congenital limb deficiency^14^.

Although the advantages of BoMIs make them appealing for use in children, there is a challenge of using BoMIs in a developmental context. Because BoMIs rely on mapping residual body movements to the control of an external device, this mapping between body movements and the control of an external device can sometimes be non-intuitive, which may limit the ability of children to use these interfaces. A recent study^15^ examined the use of a BoMI for cursor control and found that children showed lower performance (as indexed by longer movement times) than adults. In addition to task performance, there was also a difference in the coordination of the movement, with younger children showing different coordination strategies compared to adults. These results suggest that understanding the process of how children learn to control these BoMIs is an important step for successful translation in this population.

In this study, we aimed to extend these results to study how children actually control a robot arm using a BoMI. Even though the problem of robot arm control is conceptually similar to cursor control, there are two critical challenges in the control of a robot arm. First, because of the high number of DOFs (e.g., shoulder, elbow, wrist, and gripper), robot arms usually use ‘velocity-control’ (i.e. the interface controls the end-effector velocity) instead of ‘position-control’ which is typically used for a screen cursor (i.e. the interface controls the cursor position). This distinction is important because velocity control is known to be less intuitive than position control^16–18^, but on the other hand may require less movement exploration (since participants are near the ‘resting’ posture at the end of each motion, which corresponds to zero velocity). Second, there is an issue of learning to accommodate delay - unlike the cursor which is a virtual object with zero inertia that can move instantaneously, the robot arm is a physical object with inertia and associated time delays in the response. This difference may also affect the learning strategy because there is evidence that adapting to these delays in an interface can be challenging^19–21^.

The goal of the study was to characterize differences between children and adults when learning to control a robot arm using a BoMI. Instead of focusing on specific learning mechanisms (like feedback or feedforward control), our aim was to examine overall age differences in learning in this functional task, which has direct real-world relevance. We tested typically developing children, young adults, and one child with congenital limb deficiency. Based on our previous results, we hypothesized that children would show lower performance (longer movement times and path lengths) compared to adults. Moreover, based on the results from our prior study^15^ we also examined if there were differences in movement exploration between children and adults when using velocity-control.

## Methods

### Participants

A total of 48 participants volunteered for the study. Participants in this task were selected from one of three age groups: (i) 9-year olds (n = 11, age range between 8–10 yrs), (ii) 12-year-olds (n = 12, age range between 11–13 yrs), and (iii) young adults (n = 25, age range 18–25 yrs). The age groups of the children were selected based on our prior work on cursor control^15^, which showed age-differences in learning the task. In addition, one child (age 12 yr) who had congenital limb deficiency of both upper and lower limbs participated in the experiment. Children were paid $10 for their participation, and young adults (all college students), received extra course credit. All participants provided informed consent or assent (including parental consent in case of children) and experimental protocols were approved by the IRB at Michigan State University. Methods were carried out in accordance with the ethical standards of the IRB and with the Declaration of Helsinki.

### Experimental Setup

**Figure 1:**
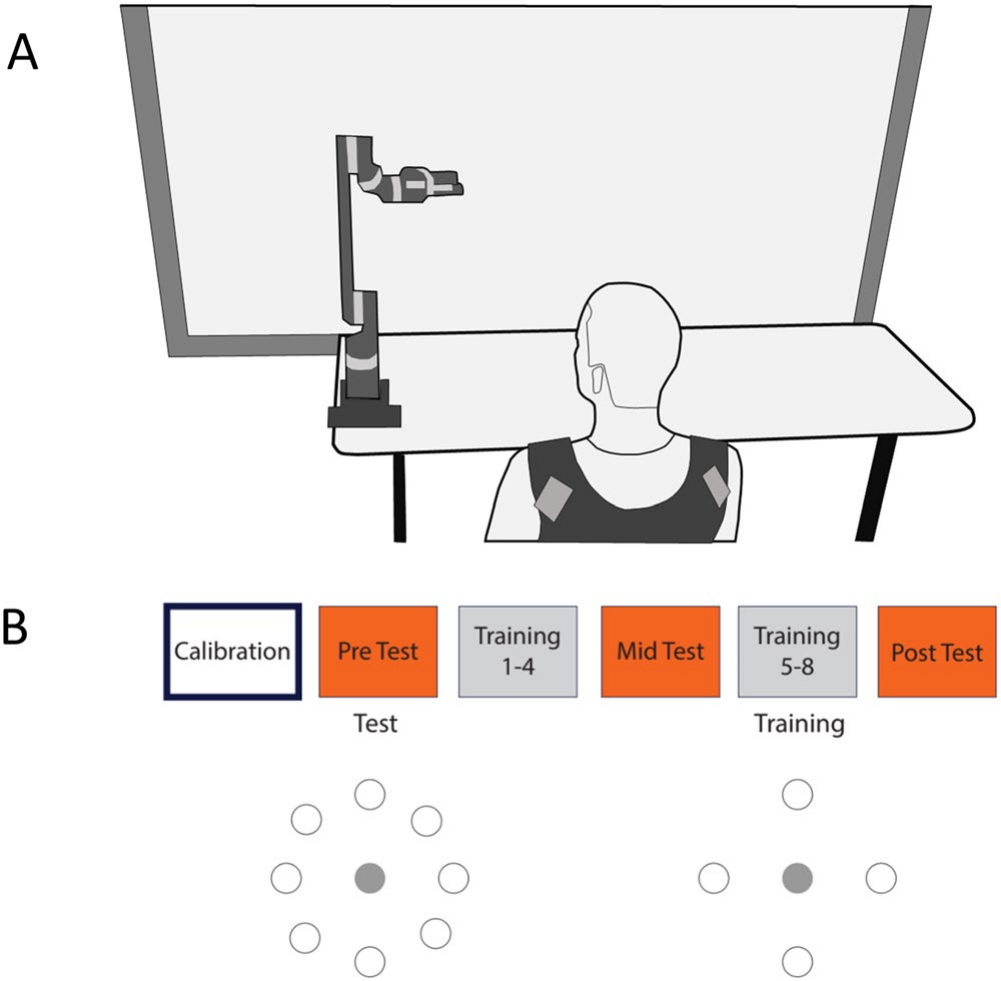
(**A**) Schematic of experiment. Participants wore inertial measurement units bilaterally near the shoulders and moved the end effector of a robot arm to targets shown on a projection screen. (**B**) Experimental protocol – all age-groups first performed a calibration phase (that determined how the IMU signals were mapped to the velocity of the robot), and then proceeded to perform two types of blocks – training and test blocks. The test blocks (which were our assessments of learning) contained 8 targets as shown, whereas the training blocks only had 4 targets in the cardinal directions.

Participants sat in front of a projection screen and had to guide the end-effector of a commercially available 7 DOF robot arm (JACO arm, KINOVA robotics, Boisbriand QC, Canada), mounted in front of the screen, to specific targets on the projection screen (Fig. 1A). The robot arm is anthropomorphic with 2 DOFs at the shoulder, 1 DOF at the elbow, 3 DOFs at the wrist, and a 1 DOF gripper (specifications of the robot can be found at www.kinovarobotics.com). Because targets were displayed on a projection screen (i.e. a planar surface), participants only controlled two dimensions of the robot end-effector (the end-effector refers to the last link – i.e. the ‘hand’ of the robot arm, so participants had to control its horizontal and vertical position to reach a specific target projected on a screen). With the exception of the robot arm, the projection screen and the mode of control (which was unique to this study), the task and procedures were similar to the cursor control task described in previous studies^14,15^ and are summarized below.

Four wireless inertial measurement units (IMUs) (YEI Technologies, OH) were attached to the participant near the acromioclavicular joint on both left and right sides of the body (both anteriorly and posteriorly) using a velcro vest. The IMUs measured roll, pitch and yaw angles, but for the purpose of this experiment we used only the roll and pitch angles. The IMUs’ sampling frequency was set at 50 Hz for the first calibration period (which did not involve control of the robot), and set to 100 Hz during the actual control of the robot.

### Controlling the robot arm using IMU signals

We used a ‘velocity-control’ mode to control the end effector by mapping the 8- IMU angles into the 2-D velocity of the robot end-effector. The mapping of the IMU signals to the end-effector velocity of the robot was achieved as follows:

$$[\begin{array}{c}{v}_{x}\\ {v}_{y}\end{array}]={\boldsymbol{A}}{[\begin{array}{ccc}{s}_{1} & {s}_{2} & \begin{array}{cc}\cdots  & \end{array}{s}_{8}\end{array}]}^{T}$$

where v_x_, v_y_ are the horizontal and vertical velocities of the end effector (in m/s), the matrix **A** is the ‘map’ that transformed IMU signals into end-effector velocities, and s_1_..s_8_ are the 8 angles from the IMUs (4 sensors × 2 angles), and the superscript T indicates the transpose of the matrix. These end-effector velocities were sent at 100 Hz directly as inputs to the robot. The robot then internally performed inverse kinematic and inverse dynamic computations for joint angle velocities and actuator torques to produce the commanded end-effector velocities in the X and Y directions while holding all others values (Z position and end-effector orientation) constant. It is important to note that this map is redundant – i.e. there are multiple motions of the body that can give rise to the same end-effector velocity.

### Protocol

#### Calibration

To determine the matrix A that mapped body motions to robot end-effector velocity, participants performed a free exploration of their upper body movements. During this phase we instructed participants to explore different motions with their upper body for 1 minute. No specific instructions were provided on what motions to perform other than to maintain a comfortable range of motion. We ran principal components analysis (PCA) on these data and extracted the first 2 principal components (PCs), and their corresponding variances. These two PCs formed the two rows of the matrix A (i.e. the x-component was controlled by motion along PC1 and the y-component was controlled by motion along PC2). The PCs were normalized, divided by the square root of their respective variance, and then multiplied by 10 m/s. This value of 10 m/s was chosen to provide reasonable velocities of the end-effector.

Because even small unintentional deviations (such as those due to breathing) from the resting posture could be captured by the IMUs and potentially affect the velocities, we implemented a ‘dead-zone’ of 0.2 m/s so that robot actually started moving only when the velocity exceeded this threshold. When the velocity exceeded this threshold, we subtracted the value of 0.2 from the magnitude of computed velocity to maintain smooth control (e.g. a computed velocity of 0.3 m/s would only cause the end effector to move at 0.3–0.2 = 0.1 m/s).

Once the calibration was completed and the mapping was determined, participants performed the protocol (Fig. 1B) consisting of 11 practice blocks in total. There were two different types of practice blocks:

#### Training blocks

In the training blocks, participants performed a center-out reaching task with the robot. The goal of the participants was to move the end effector of the robot into the specified target shown on the projection screen as quickly as possible. Targets were arranged in a circular pattern with amplitude 220 mm and radius 21.4 mm. In the training blocks, only 4 targets (along the cardinal directions) were presented. Each target was presented three times for a total of 12 trials. Trials were not time-limited – a target had to be achieved before a subsequent target was shown. Targets were presented in a pseudorandom sequence with the constraint that all targets had to be presented once before a target could repeat. There were eight training blocks in total.

#### Test blocks

Test blocks were identical to training blocks, except for the fact that there were 8 targets presented (4 targets in the cardinal directions, and 4 targets along the diagonals). Each target was presented twice for a total of 16 trials. Targets were presented in a pseudorandom sequence with the constraint that all targets had to be presented once before a target could repeat. There were three test blocks (pre/mid/post) in total.

There was no particular rationale for selecting the specific number of trials in each block other than to obtain a reasonable number of practice trials within a single experimental session (lasting 60–90 min) without inducing excessive fatigue or boredom. Also, while the task was to position the end-effector at a specified target, it is critical to note that the control was still only in the velocity domain – i.e., participants’ motions (as measured by the IMUs) controlled the velocity of the end-effector, and therefore stopping at a target could be achieved by bringing the velocity to zero.

## Data Analysis

### Task completion

To measure how many participants in each age group could complete the task, we computed a task completion rate for each block as the percentage of individuals in the age group who were able to complete the given block. A block was considered incomplete if participants either voluntarily asked for the experiment to be stopped, or if the total testing time for that block exceeded 1.5 hrs. Once a block was considered incomplete, the experiment was stopped; so all subsequent blocks were also considered incomplete.

### Movement time

Movement time was measured as the time taken from the instant that the robot end-effector left the home position to the time when the end-effector reached the target and stayed inside it for 500 ms.

### Path length

To examine the efficiency of the path chosen (which indicates the degree of control they had over the end effector), we computed a path length metric which measured the distance traveled by the end effector when moving from the home position to the target location, and normalized it by the straight-line distance between the two. Moving exactly in a straight line between the home position and target location would therefore result in a path length of 1. Higher path lengths would imply that the end-effector took a longer path to get to the target, indicating less control of the end-effector.

### Coordination analyses

To examine the movement coordination in this redundant task, we ran a PCA on the IMU signals during each block. From this we computed the variance accounted for (VAF) by the first two components - PC1 and PC2. We chose the first 2 PCs because of the fact that the task required 2 dimensions of control. From these two numbers, we computed two metrics – a ‘planarity’ metric (i.e. the sum of the VAF in PC1 and PC2) and the ‘aspect ratio’ (the ratio of VAF in PC2 to PC1). The planarity is an index of the overall movement exploration (a higher number indicates that the dimensionality of the IMU signals generated was closer to a 2-D plane) and the aspect ratio indicates the degree to which participants get stuck in one coordination pattern (a lower aspect ratio indicates a higher reliance on one coordination pattern).

## Statistical Analysis

Because the number of 9-year olds who completed the full experiment was very low (see task completion rate in Results), we dropped this group from the statistical analysis, but present these results in the graphs. All dependent variables were analyzed using a 2 × 3 (Group × Test) mixed model ANOVA, with Group (12-yr, adult) as the between-subject factor and test (pre, mid, post) as the within-subject factor. When applicable, the Greenhouse-Geisser correction was applied to correct for violations of sphericity. The significance level was set at α = 0.05. Effect sizes for ANOVAs are reported as ω^2^ values, with the corresponding interpretation - small effect (0.01), medium effect (0.06), and large effect (0.14)^22^

The data from the child with congenital limb deficiency is presented separately because of the following reasons – (i) the child had already participated in a similar experiment with a cursor control task, and (ii) we encountered some technical difficulties on his first day of practice with the robot, as a result the data presented are essentially his second day of practice at the task. Because this was a single case, no statistical analysis of these results was performed.

## Results

### Task completion rate

**Figure 2:**
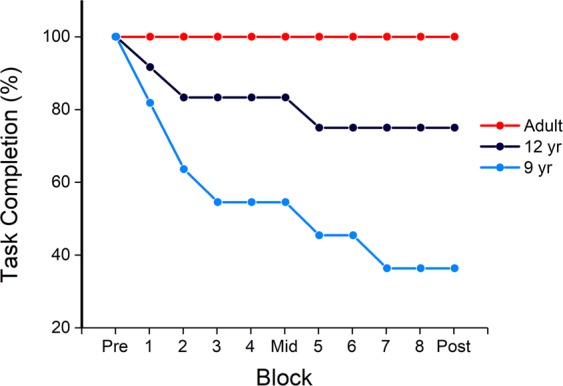
Task completion rate as a function of different age groups – the pre, mid, post indicate the test blocks, and the blocks numbered 1–8 indicate the training blocks. Younger children (9- and 12-year olds) had a lower task completion rate compared to adults, indicating greater difficulty with learning the interface.

The task completion rate for the three groups is shown in Fig. 2. The percentage of participants who were able to complete the full experiment varied differed between age groups, with the 9-year old group having the greatest difficulty with completing the task.

### Calibration analyses

In order to verify that any differences in the robot control task between children and adults was not due to any prior differences in the calibration, we performed two analyses. First, we analyzed the VAF accounted by PC1 and PC2 from the calibration phase (when they did not have to control the robot). These were not significantly different across all 3 groups, VAF PC1: Adult: 52.40 ± 8.73%, 12-yr: 55.05 ± 10.88%, 9-yr: 57.99 ± 12.74%, F (2.45) = 1.166, p = 0.321. VAF-PC2: Adult: 23.81 ± 5.32%, 12-yr: 22.58 ± 5.93%, 9-yr: 20.81 ± 7.79%, F(2,25) = 0.935, p = 0.400).

Second, to determine if the principal components themselves were systematically different between adults and children, we used a bootstrap method as follows: (i) we randomly picked one person P1 from the adult group, (ii) we randomly picked a second person P2 from either the adult, 12-year old, and 9-year old groups. We computed the angle between the principal components (i.e. PC1 of P1 with PC1 of P2, and PC2 of P1 with PC2 of P2) using the subspace command in MATLAB. We repeated this analyses 100 times, generating a total of 200 angles (100 angles for PC1, 100 angles for PC2) for each of the Adult-Adult, Adult-12-yr, and the Adult-9 yr comparisons. If the PCs were somehow systematically different between groups in the calibration phase, it would be expected that the mean angle when the two individuals are within the same group (i.e. Adult-Adult) must be smaller than the mean angle when the two individuals are from different groups (Adult-12 yr or Adult-9 yr). However we found no such group differences in the mean angle between the principal components (PC1: Adult-Adult: 63 ± 20°, Adult-12 yr: 62 ± 20° Adult-9 yr: 65 ± 18°; F(2,297) = 0.447, p = 0.640; PC2: Adult-Adult: 67 ± 19°, Adult-12 yr: 67 ± 18° Adult-9 yr: 67 ± 18°; F(2,297) = 0.024, p = 0.977). These two results (both the VAF and the mean angle) suggest that any group differences in learning cannot be attributed to differences in the calibration phase.

### Movement time

**Figure 3:**
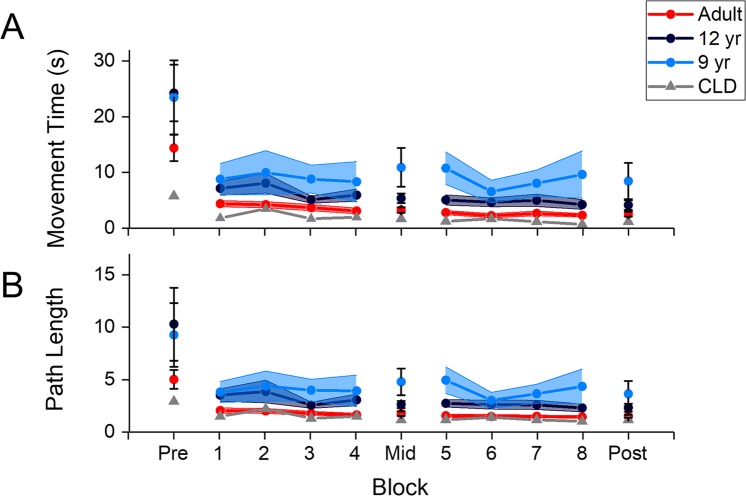
(**A**) Movement time, and (**B**) Path length for the three age groups, and the child with congenital limb deficiency (CLD) as a function of practice. Pre/Mid/Post indicate the test blocks, and 1–8 indicate the training blocks. The sudden change in performance between pre and block 1 is due to the fact that test blocks contain all 8 targets (requiring simultaneous control of x- and y-directions), whereas the training blocks only contain 4 targets (requiring only control of either x- or y-direction at a time). Both movement time and path length decreased with learning, but there were differences between age groups that were seen throughout learning. Performance in CLD was much better than age-matched controls (although see text for caveats). Only the data from participants who completed the entire experiment are presented. Error bars indicate 1 standard error.

Both the 12-year old and the adult groups showed changes in movement time with practice, but the adults showed consistently shorter movement times compared to the 12-year olds (Fig. 3A). The ANOVA revealed a significant main effect of test (F(1.05, 33.72) = 46.83, p < 0.001, ω^2^ = 0.556), and a significant main effect of group (F(1, 32) = 4.80, p = 0.036, ω^2^ = 0.101). The Test x Group interaction was not significant (F(1.05, 33.72) = 3.12, p = 0.084, ω^2^ = 0.026).

### Path length

Path length also showed a similar trend to movement time. Both groups decreased path length with practice, but the adults showed consistently shorter path lengths compared to the 12-year olds (Fig. 3B). The ANOVA revealed a significant main effect of test (F(1.03,32.98) = 22.40, p < 0.001, ω^2^ = 0.366) and a significant main effect of group (F(1,32) = 5.65, p = 0.024, ω^2^ = 0.120). The Test x Group interaction was not significant (F(1.03, 32.98) = 3.50, p = 0.069, ω^2^ = 0.043).

### Coordination analyses

**Figure 4:**
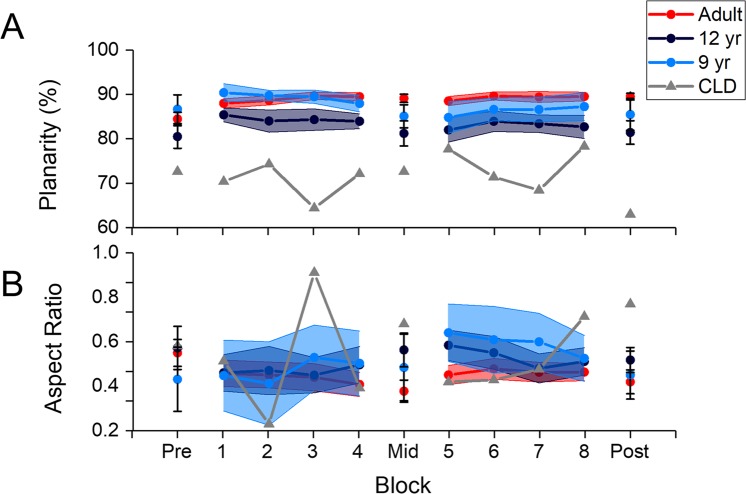
(**A**) Planarity and (**B**) Aspect ratio for the three age groups, and the child with congenital limb deficiency (CLD) as a function of practice. Pre/Mid/Post indicate the test blocks, and 1–8 indicate the training blocks. Planarity was higher in adults compared to children, indicating a more efficient search strategy, whereas the aspect ratio did not differ between the groups. CLD showed a qualitatively different pattern from the remaining groups, having a much lower planarity, and a higher aspect ratio. Only the data from participants who completed the entire experiment are presented. Error bars indicate 1 standard error.

When we examined the planarity (i.e. the sum of VAF in PC1 and PC2), we found that planarity increased with learning, but adults had significantly greater planarity than 12-year olds (Fig. 4A). The ANOVA revealed a significant main effect of test (F(1.46, 46.66) = 4.45, p = 0.027, ω^2^ = 0.088), and a significant main effect of group (F(1,32) = 10.21, p = 0.003, ω^2^ = 0.213). The Test x Group interaction was not significant (F(1.46, 46.66) = 2.22, p = 0.133, ω^2^ = 0.031).

When we examined the aspect ratio (i.e., the ratio of VAF in PC2 to PC1), there were no effects of learning or age (Fig. 4B). The ANOVA revealed that all effects were not significant – main effect of test (F(1.43, 45.61) = 2.49, p = 0.110, ω^2^ = 0.041), main effect of group (F(1,32) = 1.36, p = 0.252, ω^2^ = 0.010), and Test x Group interaction (F(1.43, 45.61) = 1.49, p = 0.236, ω^2^ = 0.013).

### Performance of child with congenital limb deficiency

The task performance of the child with congenital limb deficiency was superior (i.e., lower movement times and path lengths) relative to age-matched controls, and even most adults (Fig. 3). Planarity was also lower, whereas aspect ratio tended to fluctuate throughout practice. A comparison of the performance of CLD in the post-test against all other age groups is shown in Table 1.

**Table 1:** Mean (SD) of task performance and coordination metrics for all groups in the post-test.

	9-year old	12-year old	Adult	CLD
Number of participants who completed post-test	4/11	9/12	25/25	1/1
Movement Time (s)	8.46 (6.48)	4.15 (2.61)	2.55 (2.25)	1.21
Path length (no. units)	3.66 (2.42)	2.34 (1.14)	1.52 (0.65)	1.17
Planarity (%)	85.46 (7.16)	81.40 (7.95)	89.49 (3.65)	62.98
Aspect ratio (no. units)	0.49 (0.16)	0.54 (0.12)	0.46 (0.20)	0.72

For the 9-year and 12-year old groups, the data only includes participants who were able to complete the task. The child with congenital limb deficiency (CLD) column does not have a SD because there was only a single participant in this group.

## Discussion

The focus of the study was to examine differences between children and adults in controlling a robot arm. We examined three age groups (9-yr, 12-yr and adults) learning to control the end-effector of a robot arm in 2-D by using movements of the shoulder and torso. Our results showed a striking effect of age on motor learning - (i) the 9-year olds had the greatest difficulty with the task, with only ~40% even being able to complete the task, and (ii) compared to adults, even 12-year olds had greater difficulty in controlling the robot arm as indexed by a lower task completion rate, longer movement times and path lengths. Although the qualitative trend of children performing worse than adults at the task was perhaps unsurprising, the magnitude of the differences seen (with a majority of 9-year olds being unable to even complete the task) was somewhat unexpected.

When we examined the coordination underlying this performance of the 12-year olds, we found that they had less planarity than adults throughout practice. As mentioned in the Introduction, the 2-D task necessitates that the signal space of the participants (i.e. the IMU angles) also lie at least on a 2-D surface. The fact that 12-year olds showed less planarity indicates that they were exploring solutions that lay outside this 2-D plane, which suggests an inefficient search strategy as it indicates that they were unable to constrain their body motions to a lower dimensional space. These results, along with the path length metric, highlight that differences in movement time seen between the two groups are not simply due to biomechanical differences (like the ability to move fast), but rather reflect an underlying inability to learn and explore the appropriate solutions required for performing the task.

These results are consistent with prior studies that have shown that children have greater difficulty with learning complex and non-intuitive visuomotor transformations^23–25^. There were two levels of complexity in the current paradigm. One level of complexity arose from the fact that we mapped the end-effector velocities based on PCA, this meant that the body motions did not necessarily correspond intuitively to the motions of the end-effector. A second level of complexity arose from the fact that velocity-control of an end-effector can itself be non-intuitive (compared to position control), because even the simple motion of moving the effector forward to a target requires two opposite motions - an initial motion from the rest position (to initiate the velocity to a non-zero value in order to move from the home position) and subsequently a reverse motion back to the rest position (to bring the velocity back to zero in order to stop at the target). The time integral of motion produces the net changes in the end-effector position in the velocity-control case creating an increase in stimulus-response complexity^26^, and this may make the interface harder to learn for children. This increased complexity from velocity-control is also reflected from the fact that we found a similar trend in age-group in our previous work using position control to control a cursor, but the magnitude of age differences was much greater in the current study with velocity control.

Even when children were able to complete the task, there were marked differences in the quality of the movement (as measured by the path length). Children showed longer path lengths to get to the target, reflective of poorer planning and control. These results are consistent with prior work in reaching and grasping movements demonstrating that children have difficulties specifically in motor planning^27–29^, and require much more dependence on online feedback to make adjustments during movements. Interestingly, these behavioral results in children have also been found to correlate with electrocortical measures, which show decreased task-relevant activation of sensorimotor planning regions such as the premotor and the supplementary motor areas, and increased activation in the frontal regions^30^. Although the current study was not designed to examine these mechanisms, the increased frontal activation is consistent with the idea that children require greater attention even for relatively simple tasks such as reaching, and therefore further increasing the complexity of the novel task using the BoMI could have adversely affected their learning.

It is important to point out that although the 2-D task used in this particular experiment could have been made more intuitive for the children, intuitiveness is not always guaranteed in BoMIs because control is based on movement repertoire, which can be limited in many circumstances. This is not only true for the current BoMI, but for the class of BoMIs that rely on body motion for control (e.g., head movements). For example, if the ability to lean forward is impaired in an individual, the individual may have to learn to use another, likely less intuitive, motion to move the robot forward. Therefore, our goal was to address the general method of mapping a high dimensional body space to the control of an assistive device. In this regard, PCA has been used as an automated procedure because it provides the dimensions of greatest variance, which can then be used to identify a set of candidate movements - sometimes termed ‘synergies’ or ‘eigen movements’^10,31–33^ - for control of the assistive device. However, there are two downsides of PCA – (i) the principal components from PCA are linear combinations of movements across multiple sensor values, which in our case meant that even a simple motion of moving along one axis could potentially correspond to motions at multiple joints on the body; (ii) even though PCA guarantees that the PCs are orthogonal in a mathematical sense, that does not necessarily mean that motions along different PCs can be performed independently in an anatomical sense. In view of these disadvantages of PCA, it may be critical to explore other dimensionality reduction methods (including other variations of PCA - for example using a sparsity constraint^34^) which can yield simple candidate motions to control the interface. An alternative approach is to map control signals into the motion of a virtual body, which allows for intuitive control using anatomically distinct motion patterns^35^.

These results highlight the need for designing intuitive, easy-to-learn body-machine interfaces for children. Although previous approaches in both brain- and body-machine interfaces have shown learning of seemingly arbitrary mappings^36–40^, our results show that younger children have more difficulties with learning non-intuitive mappings. In addition to more intuitive control methods as discussed above, a parallel approach to addressing this issue is by using ‘closed-loop’ interfaces where instead of the human having to learning a fixed interface, the interface can also gradually adapt itself to the movements of the human^41–43^. This ‘dual-learning’ paradigm (i.e. the human learns the interface but the interface also learns to adapt to the human) may be critical to accelerating learning of body-machine interfaces in young children.

The child with congenital limb deficiency showed much better performance compared to age-matched peers, but there are some caveats. As mentioned in the Methods section, this comparison is partially confounded by the fact that the child was not entirely naïve to the task (having already performed a prior experiment with cursor control); however, the results seen here are similar to our prior study which involved a cursor control task, where the child showed better task performance relative to age-matched controls^14^. In addition, the child showed a qualitatively different pattern in the planarity and aspect ratio results compared to the age-matched controls and the adults. The planarity was much lower than most other individuals (indicating a high degree of exploration), and the aspect ratio was generally high (indicating that he was using a combination of different coordination patterns in order to achieve the task). Although these results raise some interesting questions regarding how the learning process in this task is influenced by the available movement repertoire, and whether the child displays greater control of the torso due to reorganization from neural plasticity^44^, they do show that the interface is suitable for the target population that would require the control of a prosthetic arm.

In conclusion, we found that children have difficulty in learning to control a robot-end effector using velocity-control with a body-machine interface. Specifically, methods of controlling assistive devices that potentially yield non-intuitive control strategies may be problematic for children even as old as 12 years. While it remains to be seen if a longer training duration is sufficient to overcome these deficits, these results highlight the importance of developing new approaches for adapting body-machine interfaces for pediatric populations.

## Data Availability

The datasets generated during the current study are available from the corresponding author on reasonable request.
